# High adiposity is associated cross-sectionally with low self-concept and body size dissatisfaction among indigenous Cree schoolchildren in Canada

**DOI:** 10.1186/1471-2431-13-118

**Published:** 2013-08-12

**Authors:** Noreen Dianne Willows, Denise Ridley, Kim D Raine, Katerina Maximova

**Affiliations:** 1 Department of Agricultural, Food and Nutritional Science, University of Alberta, Edmonton, Canada; 2 Centre for Health Promotion Studies, University of Alberta, Edmonton, Canada; 3 School of Public Health, University of Alberta, Edmonton, Canada

**Keywords:** Obesity, Body image, Self-esteem, Self-concept, Canada, First nations, Aboriginal, Indigenous, Child, Indians, North American

## Abstract

**Background:**

Obesity and mental health problems are prevalent among indigenous children in Canada and the United States. In this cross-sectional study the associations between adiposity and body size satisfaction, body image and self-concept were examined in indigenous children in grades four to six living in Cree communities in the Province of Quebec (Canada).

**Methods:**

Weight status and body mass index (BMI) z-scores were derived from children’s measured height and weight using the World Health Organization growth reference. Multivariate regression models that included child’s age and sex were used to assess the association between (a) weight status and physical appearance satisfaction using pictorial and verbal body rating measures in 202 of 263 children, and (b) BMI z-score and self-concept measured using the Piers-Harris Children’s Self-Concept Scale in a subset of 78 children.

**Results:**

Children (10.67 ± 0.98 years) were predominantly overweight (28.2%) or obese (45.0%). Many (40.0%) children had low global self-concept indicating that they had serious doubts about their self-worth and lacked confidence. About one-third (34.7%) of children did not like the way they looked and 46.3% scored low on the physical appearance and attributes domain of self-concept indicating poor self-esteem in relation to their body image and physical strength, feeling unattractive, or being bothered by specific aspects of their physical appearance. Compared to normal weight children, overweight and obese children were more likely to desire being smaller (OR=4.3 and 19.8, respectively), say their body size was too big (OR=7.7 and 30.6, respectively) and not liking the way they looked (OR=2.4 and 7.8, respectively). Higher BMI z-score was associated with lower scores for global self-concept (β=−1.3), intellectual and school status (β=−1.5) and physical appearance and attributes (β=−1.3) indicating negative self-evaluations in these areas. Despite comparable weight status to boys, girls were more likely to have lower scores for global self-concept (β=−3.8), physical appearance and attributes (β=−4.2), desiring to be smaller (OR=4.3) and not liking the way they looked (OR=2.3).

**Conclusions:**

The psychosocial correlates of obesity are important considerations for indigenous children, particularly girls, given that poor self-concept and body size dissatisfaction negatively impact mental and emotional qualities of life.

## Background

Obesity and mental health problems including depression are highly prevalent among indigenous children in Canada (i.e., First Nations, Inuit, and Métis) and the United States (i.e., American Indians and Alaska Natives) [1-4]. Among indigenous children, as in the general pediatric population, obesity and mental health problems are likely entwined, with each exerting cause and effect influences on the other [5]. Considering Western society’s positive valuation of thinness, obese children often experience social rejection and victimization by peers, and have low quality of life indicators, low self-concept and poor self-perception [6-9]. Body image is a child’s internal representation of their own outer appearance [6] whereas self-concept (also called self-esteem) is how a child feels about all the characteristics that make up his or her person, taking into account, among other things, skills and abilities, interactions with others, and physical self-image [10]. Low self-concept undermines a child’s realization of their full human potential in a range of settings given that children who do not feel good about themselves and their abilities are prone to anxiety and depression [11].

If indigenous children have internalized the negative values and perceptions of the mainstream society about obesity, then obesity not only potentially predisposes indigenous children to chronic disease but is also a stigmatizing condition which can compromise children’s mental wellbeing by threatening self-concept and creating body size dissatisfaction and poor body image [7-9]. Once self-concept and body image are internalized they tend to remain stable, underlining the importance of efforts in childhood to protect them [7-9,11]. Research among indigenous children, as with other children, has often lacked an examination of these and other important psychosocial correlates of excess adiposity [8].

The objective of the present study was to provide an understanding of the association between adiposity and body size satisfaction, body image and self-concept in Cree First Nations schoolchildren living in the Province of Quebec in Canada. The remote communities where these children live are characterized by a high prevalence of obesity and obesity-associated chronic diseases such as type II diabetes mellitus [12,13]. Virtually no data exists about body size satisfaction or self-concept in indigenous children in Canada. The specific aims of the study were to examine the relationship between (a) weight status and physical appearance satisfaction using both pictorial and verbal body rating measures, and (b) adiposity and self-concept using a validated psychometric scale.

## Methods

### Study population

Data were from the Emiyuu Ayayaachiit Awaash (Active Kids) Project that gathered information on weight status, health behaviours (dietary intake and physical activity), diabetes awareness, self-concept and body size perceptions among children in grades 4 to 6 in two Cree communities (each with population <3000 persons) in northern Quebec. Data were collected in the fall of 2004 and 2005. The study was conducted in English (a language of instruction at school) with the assistance of a Cree interpreter. Results regarding child weight status (assessed using the International Obesity Task Force growth reference [14] or the US Centers for Disease Control growth charts [15]) and diabetes awareness, body size perception and lifestyle behaviors appear elsewhere [16-19].

All children provided verbal assent to a statement by the interviewer asking if they were willing to participate and their parents provided written informed consent for their child to participate. Ethics approval was received from the Human Research Ethics Board of the Faculty of Agriculture, Forestry and Home Economics at the University of Alberta. The study was approved by the school Principals and the Research Office of the Cree Board of Health and Social Services of James Bay (Quebec) which also reviewed the manuscript.

### Measures

#### Anthropometrics

Height was measured at maximal inspiration using a set square and a standard tape measure, and weight using a portable electronic scale (Health-O-Meter Professional Scale; Model HAP300-01; Boca Raton, FL, USA). All measurements occurred in a private room with two researchers present. Children were dressed in light indoor clothing without shoes. Body mass index (BMI) was calculated to the nearest 0.01 kg/m^2^. BMI z-scores and weight status categories (normal weight, overweight but not obese, obese) were created according to the World Health Organization age- and gender-specific reference values [20].

#### Pictorial and verbal measures of physical appearance satisfaction

Three measures were used to assess children’s physical appearance satisfaction in a classroom setting. Using a paper survey, children were asked ‘What do you think of your body size?’ where the response options were ‘too big’ , ‘too small’ and ‘just right’. For the question “Do you like the way you look now?” the response options were ‘yes’ or ‘no’. Questions were read out loud to children, who then circled their responses. Culturally appropriate figures (line drawings) of eight American Indian boys and girls ranging in order from thin to obese were used to evaluate children’s perceived and desired body size. [21]. Children selected the figure that represented their own body size and the figure that represented their ideal body size using the respective questions “Circle the figure that looks the most like you” and “Circle the figure that you want to look like”. Girls chose from female figures and boys from male figures. To analyze responses, figures were numbered from one (smallest) to eight (largest). The discrepancy between the figures that children chose to represent their perceived and desired body sizes was used as a measure of body size satisfaction [22]. Children with no discrepancy between the two figures were considered to be ‘ok’ with their body size (i.e., to have body size satisfaction), children with positive discrepancy were considered to have a ‘desire to be smaller’ , and children with negative discrepancy were considered to have a ‘desire to be bigger’.

#### Psychological self-concept

Self-concept data was collected only in 2004. Children completed the Piers-Harris Children’s Self-Concept Scale, 2nd edition (PHCSCS-2) [10]. PHCSCS-2 is a self-report questionnaire prompting yes/no answers to 25 positively and 35 negatively phrased items (e.g. ‘I am smart’; ‘My looks bother me’). It measures self-concept globally as well as in six domains: behavioral adjustment, intellectual and school status, physical appearance and attributes, freedom from anxiety, popularity, and happiness and satisfaction. Higher scores indicate a more positive self-evaluation in the domain being measured [10]. The physical appearance and attributes domain is similar to body image. It measures the child’s appraisal of his or her physical appearance, as well as attributes such as leadership and the ability to express ideas. T-scores from the PHCSCS-2 of 40 to 60 represent the normal range for global self-concept and each of its domains [10].

### Statistical analysis

The association between a child’s weight status and each of the three physical appearance satisfaction measures was estimated in multivariate regression models that included child’s age and sex. Separate models were fitted to the data for each outcome measure. Specifically, multinomial logistic regression models were fitted for body size dissatisfaction based on the discrepancy between the two figures (‘ok’ as the reference category) and responses to the question ‘What do you think of your body size?’(‘just right’ as the reference category). Logistic regression model was fitted for physical appearance satisfaction based on responses to the question “Do you like the way you look now?” (‘yes’ as the reference category).

For each child, raw scores for global self-concept and its six domains from the PHCSCS-2 were converted to standardized t-scores. The association between child’s BMI z-score and t-scores for global self-concept and its six domains were estimated using separate multivariate linear regression models that included child’s age and sex. Given the smaller sample of children who completed the PHCSCS-2 BMI z-score was chosen over weight status in the models. In our sensitivity models (results not shown), the multivariate regression models also included physical activity (mean step count from pedometers worn for three consecutive days) and dietary factors (mean daily calories consumed over three consecutive days, number of fruits and vegetables consumed each day, etc.). Additional analyses also included models with interaction terms for sex and weight status to formally test sex differences in these associations. Since results remained robust across all these sensitivity models and interaction terms for sex and weight status were not statistically significant, we present results from the most parsimonious models based on the largest sample size. All analyses were carried out using Stata Version 11, College Station, TX, USA.

## Results

Of the 263 children in grades four to six in both communities, 202 (76.8%) participated in the study and had parental consent for their data to be used. Of the 151 students participating in 2004, 105 (69.5%) completed the PHCSCS-2; however, self-concept data are only reported for the 78 children without evidence of response bias (i.e., a tendency to respond yes or no irrespective of item content) or response inconsistency (i.e., random response patterns) in their answers [10]. These 78 children did not differ from the full sample (n = 202) in terms of their BMI or weight status.

Characteristics of children and their physical appearance satisfaction are in Table 1. The majority was overweight or obese. Children in the three weight classes did not differ significantly by age or gender. Results of the PHCSCS-2 are in Table 2. Many children had low scores indicating negative self-evaluation in the domain being measured, particularly for the physical appearance and attributes domain.

**Table 1:** **Characteristics of children**’**s weight status and physical appearance satisfaction (n**=**202) and psychological well**-**being (n**=**78)**

	**Full sample (n**=**202)**		**Sub**-**sample (n**=**78)**	
**Variable**	**Mean**	**SD**	**Mean**	**SD**
Age, years	10.67	0.98	10.85	1.03
BMI, kg/m^2^	23.05	5.07	23.86	5.52
BMI z-score^a^	1.78	1.22	1.90	1.23
	**n**	**%**	**n**	**%**
Girl	114	56.44	50	64.10
Normal weight^b^	54	26.73	18	23.08
Overweight^b^	57	28.22	22	28.21
Obese^b^	91	45.05	38	48.72
*Pictorial discrepancy*				
desire to be smaller	128	63.37	60	76.92
ok with body size	49	24.26	11	14.10
desire to be bigger	25	12.38	7	8.97
*What do you think of your body size*?				
too big	59	29.21	29	37.18
just right	122	60.40	45	57.69
too small	21	10.40	4	5.13
*Do you like the way you look now*?				
no	70	34.65	36	46.15
yes	132	65.35	42	53.85

^a^BMI z-scores according to the World Health Organization (WHO) age- and gender-specific reference values [20].

^b^ Weight status categories (normal weight, overweight, and obese) according to the World Health Organization (WHO) age- and gender-specific reference values [20].

**Table 2:** **Children**’**s (n** = **78) self**-**concept scores and the prevalence of low**, **average and above average scores**

	**Self concept T-score (Mean ± SD)**	**Distribution of Self concept T-scores**^a^		
		**Low range (%)**	**Average range (%)**	**High range (%)**
**Global Self concept**	42.0 ±7.3	40.0	58.0	1.3
**Self-Concept Domains**				
**Physical appearance and attributes**	39.8 ± 7.0	46.3	51.3	2.5
**Popularity**	42.1 ± 7.1	35.0	63.8	1.3
**Happiness and satisfaction**	43.2 ± 7.6	33.8	58.8	7.5
**Freedom from anxiety**	44.6 ± 8.2	30.0	60.0	10.0
**Behavioral adjustment**	44.7 ± 7.2	26.3	68.8	5.0
**Intellectual and school status**	45.2 ± 7.9	22.5	68.8	8.8

^a^Standardized scores for global self-concept fall into the following categories: <40 low, 40 – 59 average, ≥60 high. Scores for the subscales are as follows: <40 low, 40 – 55 average, ≥56 high [10].

Results from three multivariate regression models of physical appearance satisfaction based on pictorial/verbal indicators from the full sample are in Table 3. Child’s weight status was significantly and positively associated with all three indicators. Compared to normal weight children, overweight and obese children were more likely to desire to be smaller based on line drawings (OR=4.3 and 19.8, respectively), indicate their body size was too big (OR=7.7 and 30.6, respectively) and not like the way they looked (OR=2.5 and 7.8, respectively). There were also differences in physical appearance satisfaction across child’s gender and age. Girls were more likely to desire to be smaller than boys (OR=4.3), and to not like the way they look (OR=2.3). A child’s likelihood of indicating their body size was too big and not liking the way they look increased with each year of age (OR=2.3 and 1.8, respectively).

**Table 3:** **Odds ratios (SE) from regression models for physical appearance dissatisfaction (n**=**202)**

	**Model 1**			**Model 2**			**Model 3**		
	**Pictorial discrepancy**			**Think of your body size**			**Like the way you look**		
**Variable**	**OR**^**a**^	**SE**	**p-value**	**OR**^**a**^	**SE**	**p-value**	**OR**^**b**^	**SE**	**p-value**
	***desire to be smaller vs. ok***			***too big vs. just right***			***no vs. yes***		
Normal weight^c^	Ref.			Ref.			Ref.		
Overweight^c^	4.26	(2.11)	0.00	7.72	(6.34)	0.01	2.45	(1.25)	0.08
Obese^c^	19.82	(10.70)	0.00	30.61	(24.27)	0.00	7.79	(3.64)	0.00
Boy	Ref.			Ref.			Ref.		
Girl	4.25	(1.76)	0.00	1.88	(0.72)	0.10	2.32	(0.80)	0.02
Age (years)	1.29	(0.26)	0.21	2.26	(0.47)	0.00	1.84	(0.33)	0.00
	***desire to be bigger vs. ok***			***too small vs. just right***				***n/a***	
Normal weight^c^	Ref.			Ref.					
Overweight^c^	0.31	(0.19)	0.06	0.20	(0.12)	0.01			
Obese^c^	0.10	(0.11)	0.04	0.09	(0.07)	0.00			
Boy	Ref.			Ref.					
Girl	1.54	(0.84)	0.43	0.46	(0.24)	0.14			
Age (years)	0.42	(0.13)	0.00	0.66	(0.18)	0.13			

^a^ Odds ratios (OR) from multinomial logistic regression models (see Methods).

^b^ Odds ratios (OR) from logistic regression models (see Methods).

^c^ Weight status categories (normal weight, overweight, and obese) according to the World Health Organization age- and gender-specific reference values [20].

Results from multivariate regression models of psychological well-being using PHCSCS-2 in the sub-sample of 78 participants are in Table 4. BMI z-score was significantly associated with lower t-scores for global self-concept (β=−1.3), intellectual and school status (β=−1.5), and physical appearance and attributes (β=−1.3). Girls were more likely to have lower scores than boys for global self-concept (β=−3.8), and physical appearance and attributes (β=−4.2). Being older was associated with lower scores for physical appearance and attributes (β=−1.5).

**Table 4:** **Coefficients (SE) from regression models for self**-**concept measured using Piers**-**Harris Self**-**Concept Scale (2nd edition) (n**=**78)**

**Variable**	**Coeff.**^**a**^	**SE**	**p-value**
	***Global Self-Concept***		
BMI z-score^b^	−1.31	(0.65)	0.05
Boy	Ref.		
Girl	−3.78	(1.75)	0.03
Age (years)	0.02	(0.82)	0.99
	***Behavioural adjustment***		
BMI z-score^b^	−0.66	(0.67)	0.33
Boy	Ref.		
Girl	0.52	(1.80)	0.77
Age (years)	0.55	(0.84)	0.51
	***Intellectual and school status***		
BMI z-score^b^	−1.46	(0.70)	0.04
Boy	Ref.		
Girl	−3.43	(1.88)	0.07
Age (years)	−0.54	(0.88)	0.54
	***Physical appearance and attributes***		
BMI z-score^b^	−1.30	(0.59)	0.03
Boy	Ref.		
Girl	−4.15	(1.57)	0.01
Age (years)	−1.53	(0.73)	0.04
	***Freedom from anxiety***		
BMI z-score^b^	−0.97	(0.75)	0.20
Boy	Ref.		
Girl	−3.55	(2.00)	0.08
Age (years)	−0.13	(0.93)	0.89
		***Popularity***	
BMI z-score^b^	−0.35	(0.66)	0.60
Boy	Ref.		
Girl	−1.32	(1.78)	0.46
Age (years)	0.30	(0.83)	0.72
	***Happiness and satisfaction***		
BMI z-score^b^	−1.32	(0.69)	0.06
Boy	Ref.		
Girl	−2.40	(1.85)	0.20
Age (years)	−0.32	(0.86)	0.71

*Abbreviations*: BMI, body mass index (kg/m^2^).

^a^ Coefficients from multivariate linear regression models (see Methods).

^b^ BMI z-scores according to the World Health Organization age- and gender-specific reference values [20].

## Discussion

This study found that higher levels of adiposity among Cree elementary school children were associated with physical appearance dissatisfaction (i.e., children wanting to be smaller, thinking their body size was too big, and not liking the way they looked) based on both verbal and pictorial measures and low scores on the physical appearance and attributes domain of self-concept indicating poor self-esteem in relation to body image and physical strength, feeling unattractive, or being bothered by specific aspects of physical appearance [10]. These findings are concerning given that children who are unhappy with their appearance whether due to weight-related social pressures or personal weight concerns are prone to disordered eating and less likely to adopt health behaviors than children who have a positive sense of their bodies [8]. Children with greater adiposity also had lower scores for global self-concept and the intellectual and school domain of self-concept. Children with low global self-concept have doubts about their self-worth, lack confidence, and view themselves as being unpopular, having difficulty making friends and having poor relationships with family members. Children with low intellectual and school status have numerous perceived difficulties on specific school-related tasks [10].

The findings suggest that Cree children have internalized the broader societal message that thin is better and physical appearance is an important evaluative component of the self [7]. Even in remote areas of North America, children receive communications through forms of mass media such as television and the internet that influence their beliefs and attitudes about what is a healthy and optimal body size [22,23]. Cree girls had more physical appearance dissatisfaction and lower self-concept than boys despite having a similar weight status. This finding suggests girls had greater internalization of societal messages about a desirable weight or were subjected to a greater degree of criticism about their weight [22].

Strengths of this study were that children were classified into weight categories using measured rather than self-reported heights and weights. The use of ethnically appropriate line drawings to measure body size satisfaction likely allowed children to identify with the figures and choose the best response to the questions about actual and desired body size. We used a validated multi-dimensional measure of self-concept, and corroborated findings using multiple assessments that served to complement and provide independent verification of self-concept [11]. Limitations were the study’s cross-sectional design meaning the causal process between obesity and self-concept could not be discerned. Longitudinal studies are required to elucidate the nature of the relationship. Interpretation of the PHCSCS-2 may be problematic in this population. The validity of the scale with Cree children is unknown given that the normative sample was different from the children in the present study and cultural response styles can influence test results [10].

## Conclusions

There are efforts across Canada to inform First Nations people about the health risks of excess weight including the development of type 2 diabetes mellitus [1,24]. Given Cree children’s disposition for low self-concept, poor body image and body size dissatisfaction, obesity prevention efforts in Cree and other indigenous children must be cautious in their approach and take into account mental and emotional aspects of children’s well-being. When counseling about the importance of a healthy body weight care should be taken to focus on health promoting behaviors rather than solely weight loss to avoid increasing body size dissatisfaction and undermining self-worth. Although controversial, one such strategy would be the Health at Every Size (http://www.haescommunity.org/) approach which focuses on children adopting health habits for the sake of health and well-being rather than weight control [25]. It is a model which seeks to avoid the known psychological harm of stigmatizing obesity. Future obesity research in indigenous children must include a greater understanding of how obesity is perceived and its potential impact on mental wellbeing.

## Abbreviations

BMI: Body mass index; PHCSCS-2: Piers-Harris children’s self-concept scale, 2nd edition; OR: Odd ratio; SE: Standard error.

## Competing interests

The authors declare that they have no competing interests.

## Authors’ contributions

NDW and KM contributed to data analysis and interpretation. DR was responsible for data collection and data entry. KDR and DR assisted with the original research design. NDW wrote the manuscript draft. All authors read and approved the final manuscript.

## Pre-publication history

The pre-publication history for this paper can be accessed here:

http://www.biomedcentral.com/1471-2431/13/118/prepub
